# 单倍体相合高剂量非体外去T细胞外周血造血干细胞移植治疗成人Ph^+^急性淋巴细胞白血病疗效分析

**DOI:** 10.3760/cma.j.cn121090-20240411-00134

**Published:** 2025-03

**Authors:** 建丽 徐, 小飞 杜, 海龙 袁, 洪波 王, 刚 陈, 蕊雪 杨, 凯乐 张, 艾则孜 古力巴旦木, 建华 曲, 明 江

**Affiliations:** 1 新疆医科大学第一附属医院血液病中心，新疆维吾尔自治区血液病研究所，乌鲁木齐 830054 Hematologic Disease Center, the First Affiliated Hospital of Xinjiang Medical University, Institute of Hematology of Xinjiang Uygur Autonomous Region, Urumqi 830054, China; 2 河南省安阳市人民医院，安阳 455000 The People's Hospital of Anyang, Anyang 455000, China

**Keywords:** 单倍体造血干细胞移植, 急性淋巴细胞白血病, 微小残留病, Haploidentical hematopoietic stem cell transplantation, Acute lymphoblastic leukemia, MRD

## Abstract

**目的:**

分析单倍体相合高剂量非体外去T细胞外周血造血干细胞移植（haplo-HDPSCT）治疗成人Ph^+^急性淋巴细胞白血病（Ph^+^ ALL）的疗效。

**方法:**

回顾性分析2011年7月至2022年6月在新疆医科大学第一附属医院血液科接受haplo-HDPSCT模式25例成人Ph^+^ ALL患者的临床疗效。

**结果:**

25例患者中，男12例，女13例，中位年龄27（16～61）岁。23例移植前处于第1次完全缓解（CR1）状态，2例处于第2次或以上完全缓解（≥CR2）状态，微小残留病（MRD）阳性、阴性分别为8、17例。清髓性预处理21例，减低剂量预处理4例。25例患者移植后均获得造血重建，16例发生急性移植物抗宿主病（GVHD），Ⅱ～Ⅳ、Ⅲ/Ⅳ度急性GVHD累积发生率分别为（40.4±11.3）％、（4.8±4.6）％；移植后1、2年累积复发率分别为（4.0±3.9）％、（14.5±7.9）％。移植后2年总生存、无病生存率分为（81.3±8.5）％、（77.1±9.1）％。

**结论:**

haplo-HDPSCT治疗Ph^+^ ALL具有较好的疗效。

## Abstract (en)

**Objective:**

To investigate the clinical efficacy of haploidentical high-dose in vitro non-T-cell-depleted peripheral blood hematopoietic stem cell transplantation（haplo-HDPSCT）in treating adult patients with Ph^+^ acute lymphoblastic leukemia（Ph^+^ ALL）.

**Methods:**

This retrospective analysis was conducted on the clinical efficacy of 25 adult patients with Ph^+^ ALL who underwent haplo-HDPSCT from July 2011 to June 2022 at our hospital.

**Results:**

This study included 25 patients with a median age of 27（16–61）years, consisting of 12 males and 13 females. CR1 and ≥CR2 before transplantation were found in 23 and 2 cases, positive and negative minimal residual lesions were observed in 8 and 17 cases, and myeloablative conditioning and reduced-intensity conditioning were reported in 21 and 4 cases, respectively. Hematopoietic function was restored in all 25 patients after stem cell infusion. Of the 25 patients who underwent transplantation, 16 developed acute graft-versus-host disease（aGVHD）. The cumulative incidence rates of Ⅱ–Ⅳ and Ⅲ–Ⅳ aGVHD were（40.4±11.3）％ and（4.8±4.6）％, respectively. Four patients experienced relapse after transplantation, the cumulative relapse rates at 1 and 2 years after transplantation were（4.0±3.9）％ and（14.5±7.9）％, respectively. The 2-year overall survival rate after transplantation was（81.3±8.5）％ and the disease-free survival rate was（77.1±9.1）％.

**Conclusion:**

This study reveals that the unique haplo-HDPSCT protocol achieves good clinical efficacy in Ph^+^ ALL treatment.

费城染色体阳性急性淋巴细胞白血病（Ph^+^ ALL）占成人ALL的15％～30％，缓解期短、复发率高、预后较差，近年来其缓解率及预后随着酪氨酸激酶抑制剂（TKI）的应用得以明显改善[1]–[2]。目前异基因造血干细胞移植（allo-HSCT）是治愈Ph^+^ ALL患者的金标准，但移植后有20％～30％的患者复发[3]–[4]。TKI维持治疗可降低移植后复发风险[5]。本中心自2002年设计并逐步完善了单倍体相合高剂量非体外去T细胞外周血造血干细胞移植（haplo-HDPSCT）模式[6]–[9]。近年来，我们应用haplo-HDPSCT治疗25例Ph^+^ ALL患者，取得了较好的临床疗效。

## 病例与方法

一、病例

本项回顾性研究纳入2011年7月到2022年6月在新疆医科大学第一附属医院血液病中心接受haplo-HDPSCT的Ph^+^ ALL患者。本研究通过新疆医科大学第一附属医院伦理委员会审批（#20160218-129）。移植前患者或者其监护人均签署知情同意文件。

二、预处理方案

1. 清髓性预处理（ABU/CY+ATG方案）：阿糖胞苷（Ara-C）2～4 g·m^−2^·d^−1^，−9 d、−8 d静脉滴注；白消安（BU）3.2 mg·kg^−1^·d^−1^分4次静脉滴注或4 mg·kg^−1^·d^−1^分4次口服，−7 d～−5 d；环磷酰胺（CY）1.8 mg·m^−2^·d^−1^静脉滴注，−3 d、−2 d；兔抗人胸腺细胞免疫球蛋白（rATG）2.5 mg·kg^−1^·d^−1^静脉滴注，−4 d～−1 d。

2. 减低剂量预处理（FAB+ATG方案）：氟达拉滨（FLU）30 mg·m^−2^·d^−1^静脉滴注，−9 d～−5 d；Ara-C 1～2 g·m^−2^·d^−1^静脉滴注，−9 d～−5 d；BU 3.2 mg·kg^−1^·d^−1^（分4次）静脉滴注，−4 d、−3 d；rATG 2.5 mg·kg^−1^·d^−1^静脉滴注，−4 d～−1 d。

三、造血干细胞动员及采集

本组病例造血干细胞均来源于供者外周血，输注造血干细胞时单个核细胞数（MNC）一般控制为（12～20）×10^8^/kg或CD34^+^细胞≥8×10^6^/kg。

移植前4 d开始（预计采集3 d者从移植前3 d开始），供者应用G-CSF 7～10 µg/kg皮下注射。WBC>30×10^9^/L时予以阿司匹林肠溶片口服，同时限制高脂饮食。

于动员的第5、6天采集外周血干细胞，视供者情况按患者全血容量（TBV）的2～3个循环单次分离并采集外周血干细胞，并经患者中心静脉完成输注，MNC输注量（12～20）×10^8^/kg或CD34^+^细胞≥8×10^6^/kg；体重过低的供者于动员第4～6天采集或提前冻存干细胞。

四、移植物抗宿主病（GVHD）的预防、诊断及治疗

环孢素（CsA）：2.5 mg·kg^−1^·d^−1^静脉滴注，根据血药浓度进行剂量调整；霉酚酸酯（MMF）：500 mg每日2次口服，−2 d～+100 d；甲氨蝶呤（MTX）：+1 d 15 mg/m^2^静脉滴注，+3 d、+6 d、+11 d 10 mg·m^−2^·d^−1^静脉滴注；地塞米松：3 mg·m^−2^·d^−1^静脉滴注，+1 d～+30 d，白细胞恢复后改为泼尼松口服逐渐减量直至停用；抗CD25单克隆抗体20 mg/d静脉滴注，0 d（造血干细胞输注前）、+2 d。急性GVHD诊断与分级按1974西雅图诊断标准，慢性GVHD的诊断与分级按2003西雅图诊断标准。若发生急性GVHD或慢性GVHD，均加用甲泼尼龙l mg·kg^−1^·d^−1^治疗（根据情况加量或联用其他免疫抑制剂）。

五、中枢神经系统白血病（CNSL）预防

移植后常规每月给予腰椎穿刺鞘内注射甲氨蝶呤20 mg+地塞米松5 mg，未发生CNSL者连续12次。

六、造血重建标准

外周血中性粒细胞绝对计数（ANC）≥0.5×10^9^/L连续3 d视为中性粒细胞重建，PLT≥20×10^9^/L连续7 d且脱离血小板输注视为血小板重建。

七、移植后TKI的应用

移植后60 d造血重建稳定后开始使用TKI维持治疗，早期以伊马替尼（400 mg/d）等一代TKI药物为主，用药期间出现血象下降者，酌情减量至200～300 mg/d；2016年后以达沙替尼（50 mg每日2次）、氟马替尼（400 mg/d）等二代TKI为主。如果微小残留病（MRD）持续阴性，清髓性预处理方案移植患者通常维持治疗3年，若经费允许或有继续服用TKI意愿者可用至移植后5年。移植前MRD阳性或减低剂量预处理方案的患者则维持5年。

八、随访及MRD监测

移植后每月复查1次骨髓象、Ph染色、BCR-ABL基因及骨髓流式细胞术MRD，1年后每3个月复查1次，随访5年。

九、统计学处理

应用SPSS23.0软件计算患者急性GVHD累积发生率、总生存（OS）率、无病生存（DFS）率，采用Kaplan-Meier法绘制生存曲线。

## 结果

一、一般资料及移植情况

共25例患者纳入本研究。男12例，女13例，中位年龄27（16～61）岁，50岁以上患者4例。ABU/CY+ATG预处理方案21例，FAB+ATG预处理方案4例。25例患者均为单倍体供者，HLA配型检测A、B、DR 6个或A、B、C、DR、DQ 10个基因位点，配型结果≥3或5个位点者可作为供者。移植前处于第1次完全缓解期（CR1）23例，第2次或以上完全缓解（≥CR2）2例；移植前MRD阳性8例，MRD阴性17例。中位单个核细胞（MNC）输注量为14.86×10^8^/kg，中位CD34^+^细胞输注量为7.52×10^6^/kg。

二、造血重建

25例患者均获得造血重建，中位中性粒细胞植入时间为16（12～23）d，中位血小板植入时间为14（11～33）d。

三、GVHD发生情况

至随访截止，25例患者中16例发生急性GVHD，Ⅱ～Ⅳ度、Ⅲ/Ⅳ度急性GVHD的累积发生率分别为（40.4±11.3）％、（4.8±4.6）％，急性GVHD的中位发生时间为移植后45（9～91）d。肠道2例，肝脏1例，皮肤15例，口腔2例，其中累及2个部位以上4例。11例患者发生慢性GVHD，累积发生率为（44.0±9.9）％，局限型、广泛型慢性GVHD的累积发生率分别为（37.7±10.0）％、（10.1±6.8）％。

四、移植后TKI的使用

移植后23例患者使用TKI维持治疗，17例使用二代TKI，2例患者因个人意愿未使用TKI维持治疗。至随访截止，23例使用TKI维持治疗的患者中，11例疗程>3年，5例仍在服用TKI药物但随访未达3年。23例TKI维持治疗患者中有4例复发（1例移植后3个月分子学复发，调整使用TKI维持治疗后转为阴性；1例患者移植后24个月骨髓复发，接受化疗后因感染死亡；1例患者移植后12个月骨髓复发，将伊马替尼改为达沙替尼，效果不佳，后放弃治疗；1例患者移植后16个月发生分子学复发，最终全面复发并死于感染）。2例未使用TKI治疗的患者中，1例于移植后1年失访，另1例已无病生存8年。

五、移植后CNSL

25例患者均接受鞘内注射化疗（甲氨蝶呤20 mg+地塞米松5 mg）预防CNSL。1例患者在移植前诊断为CNSL，给予每周2次鞘内注射，复查脑脊液恢复正常，移植后未复发。25例患者中，4例发生移植后复发，但未发生CNSL。

六、其他移植相关并发症

25例患者中10例（40.0％）移植后发生出血性膀胱炎，经碱化尿液等对症治疗后好转。巨细胞病毒（CMV）血症1例（4.0％），带状疱疹病毒感染3例（12％），未发生EB病毒感染及移植后淋巴细胞增殖性疾病（PTLD）。17例（68.0％）患者发生肺部感染，未发生间质性肺炎。

七、生存情况

截至2022年6月6日，中位随访时间35（3～114）个月。20例患者获得无病生存，4例患者移植后复发（均为移植前MRD阳性）。移植后1、2年累积复发率（CIR）分别为（4.0±3.9）％、（14.5±7.9）％。3例患者复发后死亡，1例患者分子学复发后，经调整TKI药物再次获得持续完全缓解状态。至随访截止，5例患者死亡（3例死于原发病复发，2例为非复发死亡），移植后1、2年非复发死亡率（NRM）分别为（4.0±3.9）％、（9.3±6.4）％。移植后2年OS、DFS率分别为（81.3±8.5）％、（77.1±9.1）％。OS曲线和DFS曲线见图1。移植前MRD阳性（8例）、阴性（17例）组移植后2年OS率分别为（60.0±8.2）％、（92.3±7.4）％（*χ*^2^＝5.8，*P*＝0.015）。

**图1:**
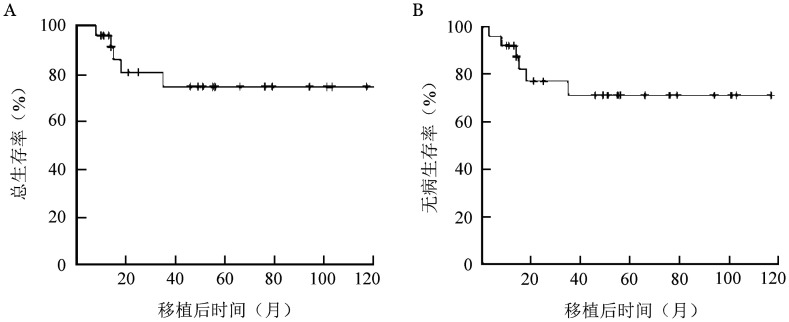
25例成人Ph^+^急性淋巴细胞白血病患者单倍体相合高剂量非体外去T细胞外周血造血干细胞移植后总生存曲线（A）和无病生存曲线（B）

25例患者的一般资料和移植情况详见表1。

**表1:** 25例行haplo-HDPSCT模式Ph^+^急性淋巴细胞白血病（ALL）患者的临床特征

例号	性别	年龄（岁）	疾病状态	供者	预处理方案	MNC输注量（×10^8^/kg）	CD34^+^细胞输注量（×10^6^/kg）	粒细胞植入时间（d）	血小板植入时间（d）	急性GVHD	慢性GVHD	移植后TKI	复发	OS期（月）
1	女	16	CR1	父亲	ABU/CY+ATG	14.88	5.23	13	16	否	否	是	是	98
2	女	18	CR1	母亲	ABU/CY+ATG	19.40	7.24	17	18	是	是	否	否	119
3	女	16	CR1	母亲	ABU/CY+ATG	20.90	5.68	13	12	是	是	是	否	15（死亡）
4	女	28	CR1	父亲	ABU/CY+ATG	20.40	10.20	13	14	是	是	是	否	102
5	女	24	CR1	母亲	ABU/CY+ATG	15.70	18.8	17	14	是	是	是	否	96
6	男	59	CR1	儿子	FAB+ATG	13.87	8.87	20	26	是	否	是	否	13
7	男	41	CR1	妹妹	FAB+ATG	11.96	5.77	17	24	是	否	是	否	8（死亡）
8	男	21	CR1	姐姐	ABU/CY+ATG	12.05	6.99	15	13	是	否	是	否	47
9	男	29	CR1	妹妹	ABU/CY+ATG	14.00	5.00	18	13	否	是	是	否	19
10	女	23	CR1	父亲	ABU/CY+ATG	20.40	16.10	17	13	否	否	是	否	48
11	女	27	CR1	父亲	ABU/CY+ATG	13.89	4.51	21	14	是	否	是	否	52
12	女	24	CR1	弟弟	ABU/CY+ATG	15.85	4.30	15	15	是	否	是	否	51
13	男	28	CR1	母亲	ABU/CY+ATG	10.44	5.21	18	33	否	否	是	否	43
14	男	21	≥CR2	母亲	ABU/CY+ATG	10.63	2.29	17	21	是	是	是	否	69
15	男	26	CR1	姐姐	ABU/CY+ATG	17.50	7.52	16	16	是	否	是	是	35（死亡）
16	男	36	CR1	妹妹	ABU/CY+ATG	14.86	8.43	23	33	否	是	是	否	67
17	女	16	≥CR2	父亲	ABU/CY+ATG	24.50	10.34	12	19	否	否	是	是	9（死亡）
18	男	24	CR1	父亲	ABU/CY+ATG	13.30	3.60	13	13	否	是	是	否	9
19	男	42	CR1	姐姐	ABU/CY+ATG	14.20	8.00	16	14	是	是	是	否	21
20	男	50	CR1	儿子	ABU/CY+ATG	10.25	10.81	16	14	否	否	是	否	13
21	男	52	CR1	女儿	FAB+ATG	15.70	13.19	13	21	是	否	是	否	12
22	女	61	CR1	儿子	FAB+ATG	16.10	15.50	18	23	否	否	是	是	42（死亡）
23	女	28	CR1	父亲	ABU/CY+ATC	13.26	11.70	14	14	是	是	是	否	10
24	女	20	CR1	父亲	ABU/CY+ATG	16.76	5.60	14	11	是	是	是	否	10
25	女	46	CR1	弟弟	ABU/CY+ATG	22.38	13.43	18	12	是	否	否	否	3

**注** haplo-HDPSCT：单倍体相合高剂量非体外去T细胞外周血造血干细胞移植；CR1：第1次完全缓解；CR2：第2次完全缓解；MNC：单个核细胞；TKI：酪氨酸激酶抑制剂；ABU：阿糖胞苷（Ara-C）+白消安（BU）；CY：环磷酰胺；ATG：兔抗人胸腺细胞免疫球蛋白；FAB：氟达拉滨（FLU）+ Ara-C + BU；OS：总生存

## 讨论

成人Ph^+^ ALL恶性程度较高，预后较差，易复发，长期生存率低[10]。TKI的问世显著改善预后[11]，第一代TKI伊马替尼及第二、三代TKI（尼洛替尼、达沙替尼、普纳替尼等）明显提高了Ph^+^ALL患者CHR率，但复发率仍较高，而且复发后再难再次获得CR，从而失去移植机会。目前国内及国外指南仍推荐，有合适供者的患者在化疗+TKI治疗获得CR后尽快行allo-HSCT[12]，移植后进行TKI维持治疗[13]–[14]。

本中心独特的haplo-HDPSCT移植模式具有以下特点：①以ATG为基础的非体外去T细胞外周血造血干细胞，MNC（12～20）×10^8^/kg或CD34^+^细胞≥8×10^6^/kg；②在常规预防GVHD模式中加用CD25单克隆抗体及低剂量短程糖皮质激素的独特移植模式，以期减少GVHD并加快造血与免疫重建、同时能很好地控制重度GVHD的发生，加强的GVHD预防措施，植入率高，且有着较好的移植物抗白血病（GVL）作用，感染发生率不高，GVHD发生率并没有明显增加[6]–[9]。通过回顾性分析25例在我中心接受haplo-HDPSCT的患者，输注的外周血造血干细胞的MNC中位数为14.86×10^8^/kg，所有患者造血功能均获重建，移植后2年OS、DFS率分别为（81.3±8.5）％、（77.1±9.1）％；移植后4例复发，移植后1、2年CIR分别为（4.0±3.9）％、（14.5±7.9）％，均为移植前MRD阳性患者；3例患者复发后死亡，1例患者分子学复发后，调整TKI后获得持续CR。较高的生存率及较低的复发率，可能输注的高剂量外周血造血干细胞有更强的GVL作用。Chang等[15]报道了30例Ph^+^ ALL患者行allo-HSCT（包括全相合移植14例，无关供者移植7例，单倍体移植9例），并在移植后接受达沙替尼治疗，3年OS、DFS率分别为76％、70.5％。意大利2019年发表的多中心研究[16]纳入441例患者（全相合移植36％，无关供者移植46％，单倍体移植15％），平均年龄44（18～70）岁，干细胞最普遍的来源是外周血（70％）。82％的患者是清髓性移植（全身放射治疗占50％，51％基于ATG），移植后40％（178/441）的患者行TKI维持治疗，移植1、2、5年的OS率分别为69.6％、61.1％、50.3％，无进展生存率分别为60.2％、52.1％、43.7％。与CR但MRD阳性的患者相比，MRD阴性患者的5年CIR较低（19.5％对35.4％，*P*＝0.001），1、2、5年NRM分别为19.1％、20.7％、24.1％。Gao等[17]分析47例Ph^+^ ALL患者haplo-HSCT的临床疗效，中位MNC输注量为7.55×10^8^/kg，中位CD34^+^细胞输注量为4.92×10^6^/kg，移植后2年OS率为63.8％，急性GVHD累积发生率为51.1％，慢性GVHD的累积发生率21.3％，CIR为19.1％。但是，高剂量移植模式在有着更强GVL作用的同时面临着GVHD的问题。本组25例患者中16例（64.0％）发生急性GVHD，其中Ⅱ～Ⅳ、Ⅲ/Ⅳ度急性GVHD累积发生率分别为（40.4±11.3）％、（4.8±4.6）％，慢性GVHD的累积发生率为（44.0±9.9）％。本组病例急性GVHD发生率并未增高，可能与我们在GVHD预防体系中加入抗CD25单克隆抗体[18]和短程小剂量糖皮质激素有关；在发生急性GVHD的患者中，重度患者占比也较低，且发生部位以皮肤为主，未出现因GVHD死亡病例，疗效较为理想。

移植前患者疾病状态对预后有重要影响，Nishiwaki等[19]回顾性研究显示：移植前MRD阴性患者的OS明显优于与MRD阳性患者，4年OS率比分别为67％、55％（*P*＝0.001）。复发率分别为19％、29％（*P*＝0.006），多因素分析显示MRD阳性是复发的重要危险因素。Spinelli等[20]研究结果显示：在移植后36个月时，移植前血液学缓解患者的OS率为80％，而PCR-MRD阳性患者为49％（*P*＝0.17），CIR分别为0％、46％（*P*＝0.027）；移植后第100天PCR-MRD阴性患者的复发率显着低于PCR阳性患者（*P*＝0.0006）。而曹星玉等[21]认为在移植前无论MRD是否阳性，Ph^+^ ALL患者移植结果相似，采用标准预处理方案和加强预处理方案预后也相似，提示即使MRD阳性也可采用标准预处理方案。本研究中，8例患者移植前MRD阳性，17例患者移植前阴性，移植后2年的OS率分别为（60.0±8.2）％、（92.3±7.4）％，MRD阴性患者较MRD阳性患者预后更好；4例复发的患者均为移植前MRD阳性，尽管高剂量外周血干细胞可产生更强的GVL作用而减少复发，但移植前MRD阴性患者可能具有更好的移植疗效。

多数研究认为移植后预防性应用TKI可改善OS和DFS并降低复发率[22]。但选用何种TKI、开始维持治疗的时机以及疗程尚无共识。Nanno等[23]在回顾性分析中，评估ponatinib维持治疗对Ph^+^ ALL患者allo-HSCT预后的影响，结果显示ponatinib维持组（9例）的2年OS率和无白血病生存（LFS）率优于非ponatinib组（100％对70.5％，*P*＝0.10；100％对50.8％，*P*＝0.02）。国内研究显示allo-HSCT后给予TKI维持治疗可显著提高疗效[24]–[25]。欧洲血液和骨髓移植组织（EBMT）建议，移植后无法检测MRD的患者应预防性应用TKI，MRD持续阴性1年后可停用TKI。≥CR2状态下接受移植的患者，应持续应用TKI，除非无法耐受不良反应；对伊马替尼耐药、有ABL激酶区突变和CNSL病史的患者，推荐应用二代TKI。Warraich等[13]发表的荟萃分析显示，TKI可改善CR1患者allo-HSCT后OS且达沙替尼疗效优于伊马替尼（尤其是对移植前MRD阳性的患者）。我中心对移植后的患者每月复查1次骨髓、Ph染色、骨髓流式细胞术MRD、BCR-ABL基因，1年后每3个月一次，随访5年，以检测预示复发的信号，在这些患者中，早期TKI治疗干预。根据患者移植后的耐受性和突变状态，使用敏感TKI进行预防性维持治疗。于移植后2个月，无特殊情况下，开始使用TKI维持治疗的有23例，取得较好疗效。由于Ph^+^ ALL易导致CNS复发，如果不进行适当的CNS预防，在整个疾病过程中CNSL的发生率会超过50％。Porkka等[26]研究发现达沙替尼在治疗颅内白血病方面具有很好的治疗潜力，对于伊马替尼中发生CNS复发的患者具有显著的临床疗效。研究显示，鞘内注射次数从8次增加到12次，可使Ph^+^ ALL患者CNSL发生率降低到0[27]–[28]。我中心移植后联合鞘内注射预防CNSL，通过给予鞘内注射甲氨蝶呤+地塞米松，每月1次连续12个月，维持治疗以达沙替尼等二代TKI为主，移植后未发生CNSL。

综上，我中心haplo-HDPSCT模式治疗Ph^+^ ALL可获得良好的造血重建及较高的生存率，可能与较高的GVL作用、移植后更长时间的TKI药物预防应用以及移植后鞘内注射预防CNSL有关。本研究样本量有限，以上结论需要进一步验证。
